# A Network Approach to Understanding the Role of Executive Functioning and Alpha Oscillations in Inattention and Hyperactivity-Impulsivity Symptoms of ADHD

**DOI:** 10.1177/10870547241253999

**Published:** 2024-05-26

**Authors:** Juan Diego Vera, René Freichel, Giorgia Michelini, Sandra K. Loo, Agatha Lenartowicz

**Affiliations:** 1 University of California, Los Angeles, USA; 2 University of Amsterdam, The Netherlands; 3 Queen Mary University of London, UK

**Keywords:** ADHD, network analysis, executive functioning, alpha oscillations, EEG, IDA

## Abstract

**Objective:**

ADHD is a prevalent neurodevelopmental disorder characterized by symptoms of inattention and hyperactivity-impulsivity. Impairments in executive functioning (EF) are central to models of ADHD, while alpha-band spectral power event-related decreases (ERD) have emerged as a putative electroencephalography (EEG) biomarker of EF in ADHD. Little is known about the roles of EF and alpha ERD and their interactions with symptoms of ADHD.

**Method:**

We estimated network models of ADHD symptoms and integrated alpha ERD measures into the symptom network.

**Results:**

EF emerges as a bridge network node connecting alpha ERD and the hyperactivity/impulsivity and inattention symptoms. We found that EF most closely relates to a subset of symptoms, namely the motoric symptoms, “seat” (difficulty staying seated), and “runs” (running or climbing excessively).

**Conclusions:**

EF functions as a bridge node connecting alpha ERD and the ADHD symptom network. Motoric-type symptoms and EF deficits may constitute important nodes in the interplay between behavior/symptoms, cognition, and neurophysiological markers of ADHD.

ADHD is a developmental disorder characterized by inattention, hyperactive or impulsive behavior, or varying combinations of these symptom domains (American Psychiatric Association, 2013; Bell, 2011). ADHD is clinically heterogeneous, likely due to multiple etiologic pathways, and high rates of psychiatric comorbidity are common. Estimates suggest that 33% to 50% of children with ADHD exhibit deficits in executive functioning (EF), such as working memory, response inhibition, planning, and vigilance (Biederman et al., 2004; Loo et al., 2007; Nigg et al., 2005). EFs are significantly associated with ADHD severity and may play an essential role in explaining the intra-individual differences in symptoms and symptom improvement in ADHD (Willcutt et al., 2005). Previous studies have found that EF is an important moderator for behavioral and stimulant medication treatment outcomes, highlighting the practical importance of understanding EF in the ADHD population (Fosco et al., 2021; Ramos-Galarza & Pérez-Salas, 2021).

## Alpha Modulation in ADHD and the Putative Role of EF as a Mediator

The alpha frequency band (8–12 Hz), a brain oscillation measured using electroencephalography (EEG), is strongly associated with top-down EF (Foxe & Snyder, 2011; Klimesch et al., 2011; Mathewson et al., 2011). Studies have found that when participants are asked to do attention or working memory tasks, alpha oscillations block task-irrelevant pathways in the brain by selectively inhibiting specific brain regions (Jensen & Mazaheri, 2010). Given the link between EF and ADHD, there are numerous studies that have investigated differences between alpha modulation in the ADHD and typically developing (TD) children (Cañigueral et al., 2022; Guo et al., 2022; Jensen & Mazaheri, 2010; Lenartowicz et al., 2019). In general, these studies have demonstrated that modulation of alpha oscillations during working memory trials is weaker in the ADHD group in comparison to the TD controls. For example, a study by Lenartowicz et al. (2014) found robust group differences in the modulation of alpha-band power during the encoding and retrieval phases of a spatial working memory task between TD controls and children with ADHD. Differences in alpha event-related decrease (ERD) between an ADHD sample and typically developing controls are not exclusive to children and are also found across the age span (Fosco et al., 2021; Michelini et al., 2022; Ramos-Galarza & Pérez-Salas, 2021). Overall, weaker alpha power modulation in ADHD samples is consistent with an altered neurocognitive system contributing to difficulties in attentional shifting and engagement during stimulus processing (Herrmann & Knight, 2001). The dorsal attention network constitutes one core element of this neurocognitive system that directly affects EF performance (Szczepanski et al., 2013).

Although prior studies have established a significant relation between EF and alpha modulation in both ADHD and TD samples, no study has yet examined how alpha power and EF interact with the individual symptoms of ADHD. Mental disorders, such as ADHD, can be conceptualized as networks of individual symptoms influencing each other (Borsboom & Cramer, 2013; McNally, 2016; van Borkulo et al., 2015). Conceptualizing ADHD as a network of symptoms and examining the effect of alpha power and EF on the symptom network can highlight probable pathophysiological pathways and contribute to targeted symptom-specific intervention efforts. The first goal of the current study is to examine the associations among the 18 DSM-5 individual ADHD symptoms and the relative contributions of each individual symptom in the ADHD symptom network. Our second goal is to integrate brain markers of attention and EF measures into the ADHD network of symptoms and explore which symptoms of ADHD are most associated with alpha ERD and EF.

A robust statistical framework for understanding relationships and interactions between symptoms of mental disorders and related covariates is network analysis (NA; e.g., Borsboom & Cramer, 2013; McNally, 2016). The network theory conceptualizes mental disorders as systems of causally connected symptoms instead of effects of a latent disorder (Borsboom & Cramer, 2013). Network analysis refers to a toolbox of statistical approaches (Freichel, 2023) that identify conditional pairwise associations among all variables included in the network. A main advantage of using network analysis in this study is that we can visualize the multivariate correlational structure between ADHD symptoms, EF, and alpha modulation; and examine which variables may significantly influence all other variables in the network. Network analysis is in line with our purpose of exploring individual ADHD symptoms instead of sum scores. Prior studies focusing on the relationship between ADHD and neurocognitive markers, conceptualize ADHD as a categorical construct with symptoms of inattention and hyperactivity-impulsivity. However, our study aims to identify associations with individual ADHD symptoms that are typically hidden in categorical diagnoses or sum-score analyses. This symptom-specific approach is congruent with current efforts in the domain of precision psychiatry to identify unique ADHD features that can be used to personalize treatment and diagnosis (Buitelaar et al., 2022).

## Network Analysis Studies on ADHD

To the best of our knowledge, only a handful of studies examined networks of ADHD symptomatology. These studies have primarily highlighted the differences across ages in the structure of symptom networks and the heterogeneity of ADHD. Martel et al. (2016) found that ADHD symptoms change with development, although symptoms such as “often easily distracted” and “difficulty sustaining attention” remained central across ages. Silk et al. (2019) used network analysis for ADHD symptoms for participants with ADHD and controls (aged 6–8 years). Their results highlighted the relative importance of the “motoric”-type symptoms in the hyperactive symptom domain.

The current study aims to first construct a network analysis for the symptoms of ADHD that will replicate prior reports. Second, in an exploratory fashion, we aim to integrate EF and alpha ERD, a neurophysiological feature of selective visual attention, into the ADHD symptom network.

## Methods

### Participants

The sample consists of 828 children (*n* = 660 ADHD, *n* = 168 TD controls) aged 6 to 18 years old who were recruited to participate in two ADHD research studies. Both samples were combined, and comparable measures were harmonized. One sample was from the baseline visit (i.e., before medications were started) of a clinical trial of ADHD medications (Loo et al., 2016), and the other sample was recruited to participate in a family study on the genetics of ADHD (Loo et al., 2010). The samples did not overlap (respective *n* = 421 and 407), and a more detailed description of the clinical and sociodemographic characteristics of the two samples can be found elsewhere (Loo et al., 2010, 2016). A mixed sample, including individuals with and without an ADHD diagnosis, was deemed suitable for two reasons: First, it provided the necessary variability across all measured variables, essential for estimating symptom network models. Secondly, our study aimed to explore the continuous associations between varying levels of ADHD symptomatology, executive functioning, and alpha ERD. Participants were recruited from the community through targeted advertisements (newspapers, television, radio, posters), primary care doctors, and local schools in the area. Participants were then briefed on the study requirements and received verbal and written consent forms approved by the local institutional review board. Loo et al. (2018) report a complete description of study protocols.

### Procedure

All participants were evaluated for ADHD (any subtype) and other childhood psychiatric disorders based on Kiddie-Schedule for Affective Disorders and Schizophrenia-PL (K-SADS-PL; Kaufman et al., 1997) and a clinical interview. Subjects were excluded if they showed any neurological disorder, head injury resulting in a concussion, diagnosis of schizophrenia or autism, or estimated Full-Scale IQ <70. Subjects were asked to refrain from taking ADHD medications for 24 hours before their visit.

### Measures

#### Clinical Measures

ADHD symptoms were measured with the parent-rated Strengths and Weaknesses of ADHD symptoms and Normal Behavior scale (SWAN) and the Swanson, Nolan, and Pelham (SNAP) rating scale, depending on the study. The SNAP and SWAN scales were harmonized to integrate both study samples (see Supplemental Table 1). SNAP responses are on a 4-point scale (0 = not at all to 3 = very much), while SWAN scores are on a 7-point scale (0 = far above average to 6 = far below average) (Swanson et al., 2012). To harmonize the two scales, we converted all SNAP scores to the SWAN metric (from 0 to 3, 1 to 4, 2 to 5, and 3 to 6). The final symptom scale for the network analysis is a 7-point scale where 0 means that the participant does exceptionally well in a behavior and a score of 6 means that the behavior is problematic. For more information on the harmonization of both scales, see Supplemental Information Section S1. The Child Behavior Checklist (CBCL 6-18; (Achenbach & Edelbrock, 1991; Achenbach et al., 2001) is a widely used parent-completed behavior rating scale that assesses a wide range of behaviors. Participants’ IQ was derived with the vocabulary and block design subtests of the age-appropriate Wechsler intelligence scale (WISC/WAIS; Wechsler, 1999).

#### Executive Functioning Latent Construct

Because the sample for this analysis comes from two different studies, where each study used different psychometric tools to measure EF, we used the integrative data analysis (IDA) framework to harmonize the two study’s EF measures and then we created a latent score for EF. IDA is an analytic tool that allows researchers to combine raw data across independent studies in a way that controls for differences across studies and provides more accurate measurement of the latent construct (Hussong et al., 2013). The EF construct was composed of seven measures across both studies, which includes the Trail Making Test, the Stroop Color and Word Tests, and the WISC/WAIS digit span subtest. Only raw scores from these scales were used to create the EF factor score. Higher scores in this construct reflect better EF (see Supplemental Information for more details on the harmonization procedure). The analysis were conducted using Mplus 8 (Muthén & Muthén, 2012) and R (Team, 2019).

#### EEG Recording

EEG was recorded using 40 Ag/AgCl electrodes, and linked ears were used as a reference. Further details on the EEG measurement are provided in (Loo et al., 2018). We used independent component analysis (ICA, Jung et al., 2000) to retrieve meaningful independent components (ICs) that reflect brain activity in midoccipital and midfrontal regions. We divided post-stimulus event-related power by a baseline (i.e., pre-stimulus window), which was subsequently log-transformed to decibel units. The baseline time period used was −600 to −100 ms. We extracted occipital alpha event-related power and then averaged across the alpha spectrum (8–12 Hz) during the encoding phase of the Sternberg spatial working memory task (0–2 seconds). Lower alpha values signify stronger (i.e., more negative) alpha ERD. For a more detailed reference for the processing steps, please see Lenartowicz et al. (2014). Participants performed a Sternberg spatial working memory (WM) task while EEG data were collected (Sternberg, 1966). Each trial began with a fixation cross shown for 0.5 seconds, then yellow dots appeared on the screen for 2 seconds, and participants were instructed to remember their location (encoding phase). Working memory load was manipulated through the number of dots shown (1, 3, 5, and 7), with more dots expected to increase working memory demands (“load”). Next, during the maintenance phase, a black screen was shown for 3 seconds and this was followed by a single-dot probe stimulus (3 seconds). Using a button press (left or right arrow key), participants were instructed to indicate whether the probe stimulus was in the same location as one of the encoding stimuli (retrieval phase).

### Data Analysis

The data analyses included two steps. First, we identified the symptoms that are most influential in the symptom network constellation. We extracted indices of the symptom importance, also called centrality indices, in the estimated symptom networks for the entire sample and separately in the networks for both groups, ADHD and typical developing (TD) groups. Second, we expanded upon the symptom networks by integrating measures of both EF and EEG alpha-ERD which are described in Supplemental Figures 2 and 3.

#### Network Estimation

We estimated four separate network structures that contained the following nodes: (1) All 18 ADHD symptoms (see Table 1), (2) inattention symptoms and occipital alpha power and EF, (3) hyperactivity-impulsivity symptoms and occipital alpha power and EF, (4) all ADHD symptoms, occipital alpha power, and EF. All networks were estimated on the entire sample (*N* = 828). The networks were estimated using the bootnet package (Epskamp & Fried, 2015) that implements the “EBICglasso” algorithm. Following visual inspection (see Supplemental Figure 4), we treated the symptoms as continuous variables. The network structure represents regularized partial correlations with an edge (edge weights = regularized/shrunk partial correlations) representing the association between two nodes after controlling for all other information in the network. Due to the large number of associations in a network and the potential for overfitting the “EBICglasso” algorithm is used. EBICglasso uses the least absolute shrinkage and selection operator (LASSO) regularization method to penalize the coefficients and avoid overfit and false-positive findings in the networks. To enforce higher specificity, thresholding in the EBIC computation was used. We set the EBIC tuning parameter to 0.5 to remove non-significant edges and avoid spurious edges. This conservative tuning parameter was considered appropriate as it errs on the side of caution (Epskamp & Fried, 2018).

**Table 1.** Demographic and Clinical Characteristics of Our Sample.

	Control (*N* = 168)	ADHD (*N* = 660)	Full sample (*N* = 828)
Sex			
Female	53 (31%)	146 (22%)	199 (23%)
Male	115 (69%)	515 (78%)	630 (77%)
Participant age (in years)	10.095 (2.752)	9.871 (2.859)	9.917 (2.838)
CBCL internalizing symptoms Mean (*SD*)	46.337 (10.053)	57.229 (10.073)	54.711 (11.057)
CBCL externalizing symptoms Mean (*SD*)	46.467 (9.137)	60.186 (10.316)	57.015 (11.595)
Estimated Full scale IQ standard score Mean (*SD*)	108.246 (13.542)	106.462 (15.515)	106.825 (15.144)

To examine the robustness and accuracy of the estimated edge weights and centrality/clustering indices, we used a case-dropping bootstrapping technique (1,000 boots), which iteratively drops an increasing proportion of observations and examines the correlation of the original estimates to those of the subsets (Epskamp et al., 2018). The correlation-stability (CS) coefficient (Epskamp et al., 2018) describes the results of the bootstraps, specifically the proportion of data that can be dropped to retain a correlation of .7 with the estimated centrality coefficients (with 95% uncertainty).

All network structures were visualized using the *qgraph* package (Epskamp et al., 2012). The network visualizations include the symptoms as nodes that are connected through lines (edges), that represent their regularized partial correlations. The thickness of the edges represents the strength of association, and the color of the edges describes the sign of the relationship (i.e., positive in blue and negative in red). All analysis scripts can be accessed through the Open Science Framework (tinyurl.com/nv4hdpwk).

#### Centrality and Clustering Indices

To quantify the relative importance of nodes in the network, we examined the connectedness and topology of symptoms in the network. We estimated *strength centrality—*commonly used centrality metric that describes the sum of all associations of a given symptom to all other nodes (Opsahl et al., 2010), thus describing the degree to which that node is implicated in the network. For instance, a node with a high strength centrality shows strong connectivity to all other nodes, and thus the node plays a significant role in the network. We refrained from estimating other popular characteristics of node importance, including betweenness and closeness, due to their potentially limited reliability in cross-sectional networks (Bringmann et al., 2019). To quantify the degree to which nodes act as bridges (“bridge symptoms”) between different communities of nodes, we estimated a measure of bridge (strength) centrality using the *networktools* package (Jones, 2018). Bridge centrality is defined as the sum of edge weights between a node and other nodes from a separate community.

## Results

### Participant Characteristics

All participants were between 6 and 18 years old and predominantly male (see Table 1). The ADHD and control groups significantly differed in gender (χ^2^(1) = 4.892, *p* = .027). There were no significant group differences with respect to age (*df* = 826, *p* = .3612, *t* = 0.914) and IQ (*p* = .1744, *t* = 1.359); however, individuals in the ADHD group reported more internalizing (*t* = −9.099, *p* < .001) and externalizing (*t* = −11.472, *p* < .001) problems.

### Symptom Networks and Centrality Measures

**Figure 1.**
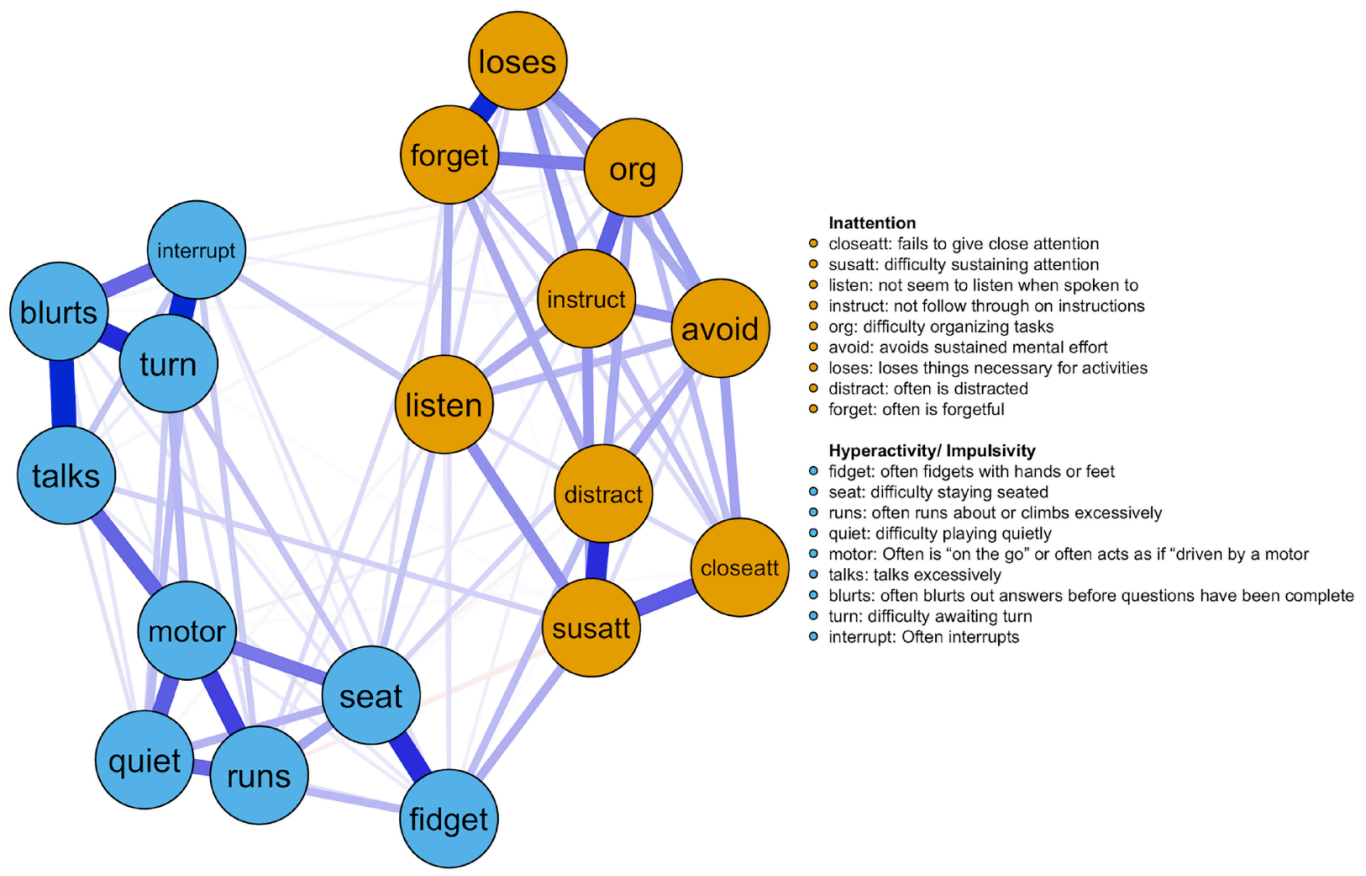
ADHD symptom network in entire sample. *Note*. Nodes are colored according to the domain that they belong to (orange = inattention, blue = hyperactivity-impulsivity). The thickness of the edges denotes the strength of association, and the color of the edges describes the direction of association (blue = positive, red = negative).

The estimated ADHD symptom network model for the entire sample is shown in Figure 1. The nodes represent the 18 ADHD symptoms, and they are colored according to the corresponding subscale (blue = hyperactivity-impulsivity, orange = inattention). The ADHD symptoms in the networks clearly separate and cluster into the inattentive and hyperactivity/impulsivity subscales. The hyperactivity/impulsivity symptoms appear to be more strongly interconnected with two separate clusters that represent verbal (e.g., items *blurts, interrupt, turn, talks*) and motoric (i.e., items *motor, seat, runs, fidget*) behaviors. Importantly, it appears that restless behavior (i.e., *fidget, seat, interrupt*) acts as a bridge between inattention and hyperactivity/impulsivity symptoms. This visual inspection is consistent with estimates of bridge centrality shown in Supplemental Figure 1. All edge weights of the networks are also provided in Supplemental Tables 3 to 6.

**Figure 2.**
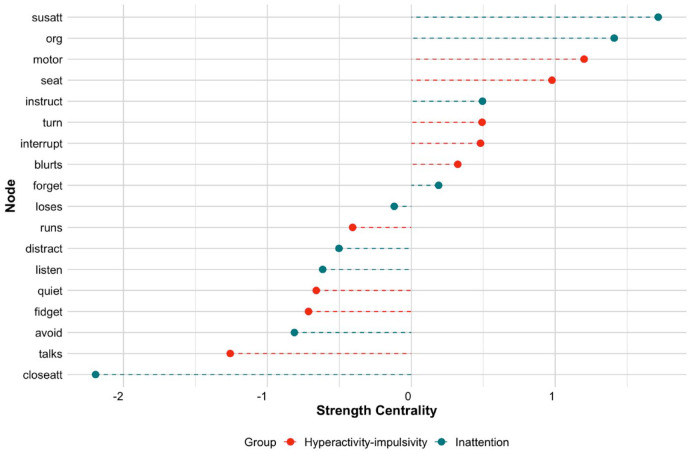
Strength centrality measures for the symptom network in the entire sample. *Note.* Standardized *z*-scores strength centrality estimates are shown. Higher scores represent higher centrality estimates and greater importance in the network.

The derived centrality measures from the symptom network are presented in Figure 2. Consistent with our visual inspection of Figure 1, several nodes show high strength centrality. Sustained attention (susatt) and difficulties organizing tasks (org) are the most central inattentive symptoms. Restless-motoric behavior (e.g., motor, seat) is most central to the hyperactive-impulsive symptom domain, while symptoms “blurts,” “interrupt,” and “turn” were also central. In general, hyperactive-impulsive symptoms show on average a stronger influence or connection to the wider network (strength centrality) than inattentive symptoms (hyperactivity-impulsivity: *M* = 0.24, *SE* = 0.20; inattention: *M* = −0.24, *SE* = 0.26).

### Integrating Alpha Oscillations and Executive Functioning into Symptom Networks

**Figure 3.**
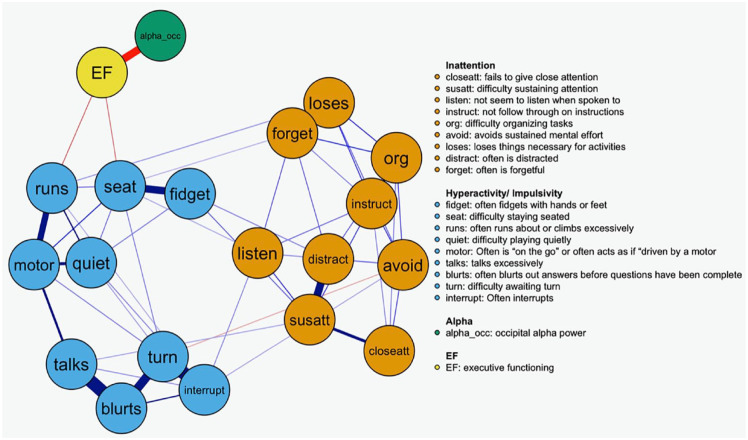
Joint symptom—EF—alpha network. *Note.* Higher symptom scores indicate stronger symptom severity/impairments. Lower alpha values signify stronger alpha ERD.

To examine how power in the alpha EEG frequency band (8–12 Hz) and EF interact with the inattention and hyperactivity-impulsivity symptom structure of ADHD, we integrated additional markers of the EF construct and occipital EEG alpha power as nodes in the network. We were interested in determining which attention and hyperactive-impulsive symptoms are most influenced by alpha and EF. Figure 3 shows EF and alpha as nodes in a joint symptom-brain-control network. The EF node is negatively associated with alpha and acts as a bridge node connecting alpha to hyperactivity-impulsivity symptoms, specifically motoric symptoms (i.e., *runs, seat*). Although the effect of the relationships between alpha and ADHD symptoms are small, they have adequate power due to the large sample size and they reflect actual underlying correlations (see Supplemental Material 4).

The network with EF, alpha, inattentive, and hyperactive-impulsive symptoms shown in Figure 3 demonstrates that EF is a bridge component connecting alpha power and the hyperactivity/impulsivity and inattention symptoms. Additional robustness analyses using network comparison tests revealed that the network structure was independent of age and sex (see Supplemental Material 6). One of the disadvantages of including the complete ADHD symptom scale is that any small to moderate effect is opaqued by the strong correlation between ADHD symptoms, and we lose some of the insights that are gained when separating by ADHD subscales. This problem is particularly pertinent when aiming to uncover cross-modal links that are typically weaker (Blanken et al., 2021; Freichel et al., 2023). We therefore estimated separate networks for the inattention and hyperactivity-impulsivity subscales—see Supplemental Material 7. These analyses replicate the findings (1. EF as a bridge node, 2. associations between symptoms *runs, seat*, and EF) shown in the entire ADHD symptom network.

### Robustness and Network Stability Analysis

We used case-dropping bootstrap (dropping rows from the data) to examine the robustness and accuracy of the estimated edge weights and centrality indices from all the previous networks. A case-dropping bootstrapping analysis (1,000 bootstraps) revealed high stability of both edge weights and strength centrality estimates. See Supplemental Figures 2 and 3 for visualizations of the stability estimates for the symptom network estimated for the entire sample. All CS coefficients can be found in Supplemental Table 7 in the Supplement Material 6. It is recommended that the CS coefficient should be above 0.25 (Hevey, 2018), thus indicating sufficient robustness and stability.. An exploratory sensitivity analysis (see Supplemental Material 6) using age groups based on median split showed no significant differences in the global ADHD symptom network structure (*p* = .428, *M* = 0.201) or global network strength (*p* = .831, *S* = 0.042).

## Discussion

Our study extends previous ADHD symptom network studies in five ways: (1) we constructed ADHD networks based on continuous ratings of the 18 ADHD symptoms from the SWAN rather than dichotomous ratings of the DSM criterion symptoms; (2) our sample consisted of a wide age range of participants from 6 to 18 years old, ADHD and controls; (3) through data harmonization, we constructed one of the largest samples to date of children with ADHD and non-ADHD (control) participants, which is relevant given the heterogeneity of symptoms in this population (Loo et al., 2018); (4) we presented the first study to integrate brain markers of attention, and EF measures into a symptom network model to examine the complex interactions with ADHD symptomatology.

The first goal of our study was to estimate a network constellation for ADHD symptoms. Overall, the ADHD symptom network in Figure 1 shows that the restless-motoric behavior (i.e., fidget),”interrupt,” “sustain attention,” and “listening” symptoms are important bridges between the inattentive and hyperactive/impulsive domains. We refer to these group of symptoms as bridge symptoms because they connect the two ADHD domains. Bridge symptoms have been associated with a higher risk for simultaneous impairment in both symptom domains (Jones et al., 2021; Kaiser et al., 2021). These critical symptoms may also be considered early warning signals and potential targets for intervention efforts (Jones et al., 2021). However, there is mixed evidence on the clinical utility of central symptoms when they are derived from cross-sectional network analyses (Bringmann et al., 2019; Rodebaugh et al., 2018; Spiller et al., 2020). For instance, McNally (2021) describes that central symptoms (in cross-sectional data) may act as either the origin or the recipient of activation, making it challenging to interpret them as treatment targets.

Figure 1 also demonstrates that symptoms of sustained attention, difficulty organizing tasks, talks excessively, and fidgeting were most central to the network. These group symptoms high on centrality exert a strong influence on the presence of other ADHD symptoms, and they may be possibly represent targets for treatment as they offer a high prognostic utility (Elliott et al., 2020). In general, we found restless-motoric behavior and sustained attention to be highly important in the ADHD symptom network, our findings are consistent with Silk et al. (2019) and Martel et al. (2016).

A novel contribution of our study is the simultaneous integration of EF and occipital alpha power into the symptom networks of ADHD. Existing studies that integrate measure of EF in symptom networks (Karalunas et al., 2021) exist, however, to the best of our knowledge, this is the first study to integrate cognitive and neural markers into networks of ADHD behaviors. We examined the role of behavioral measures of EF in the symptom network. We examined each symptom domain separately before estimating a network with all symptoms, which allowed us to highlight any small effects of the brain-behavior correlates that might otherwise be opaque by the strong correlation between symptoms. The network in Figure 3 shows that alpha enters the attention-related symptom network through EF, which closely relates to one key symptom (*listen*). Previous ADHD research in listening ability has highlighted the correlation between low EF and listening difficulties before. For example, McInnes et al. (2003) demonstrated that listening skills were significantly correlated with both verbal and spatial working memory, and parent–teacher ratings of inattention and hyperactivity/impulsivity. This link between EF and listening is expected given that listening to directions requires several executive functions such as self-control, sustained attention, and inhibition.

The network in Supplemental Figure 3 shows the relationship between the EF construct, alpha ERD, and the hyperactive/impulsive symptom network. In this network, the same pattern is replicated, where alpha ERD enters the symptom network through EF. Supplemental Figure 1 also shows that two hyperactive/impulsive key symptoms (*runs* and *leaves seat*) are bridge symptoms to the hyperactive/impulsive symptom network. We investigated the role of age considering its association with the developmental decline in the importance of hyperactivity over time (Hart et al., 1995). However, after controlling for age, we still observed a link between EF and the two bridge symptoms (runs and leaves seat).

## Caveats and Conclusion

The findings of our present study should be interpreted in light of several limitations. First, we used a composite measure of EF that may reflect a variety of different processes, including inhibition, working memory, attentional shifting, and updating. Thus, different facets of EF may show distinct interaction patterns with alpha ERD and ADHD symptoms. In contrast, the assessment of ADHD symptoms relied on parent reports that may naturally be biased toward hyperactive and impulsive symptoms, which are easily observable and tend to be more impairing than inattentive symptoms. Another concern is that our sample was predominantly male. Previous research on gender differences in ADHD has shown that females, on average, show greater improvements in EF over time, which are associated with declines in ADHD symptoms (Miller et al., 2013). More research is needed to understand gender differences in the ADHD symptom network, and which specific symptoms are most influenced by EF improvements. Lastly, to integrate data from two different studies, we harmonized the SWAN and SNAP scales (see Supplemental Material 1). This process of combining information from scales with distinct psychometric properties likely introduced measurement errors and skewed distributions that may have biased our results. For instance, the SNAP-derived scores only represent the lower end of the SWAN scale, leading to a positive skew in the harmonized dataset. The effect of skewness on network estimation remains poorly understood (Fried et al., 2015), and thus, it may have impacted the accuracy (i.e., specificity and sensitivity) of the present edges. A potential consequence of the limited variability may be the underestimation of cross-construct connections (i.e., between alpha, executive functions, and ADHD symptoms).

In general, follow-up studies should aim to investigate ADHD symptom networks in a longitudinal framework to learn more about how improvements as EF and alpha modulation may change the symptoms network across different age stages from early childhood to adulthood. Analyses using longitudinal data could further disentangle temporal (directed time effects), contemporaneous (associations at one-time point), and between subjects’ effects (Epskamp, 2020) in the network structure, and provide greater insight into the heterogeneity of ADHD. Moreover, idiographic network analytical approaches may help identify person-specific treatment targets that may potentially be leveraged in clinical practice. Future studies should also investigate the association between other frequency bands delta (1–4 Hz), theta (4–8 Hz), beta (13–30 Hz), and gamma (>30 Hz) and specific ADHD symptoms. Our study extended existing findings on networks of ADHD by integrating EF and alpha oscillatory activity. The complex interactions within ADHD symptoms and between symptoms and EF suggest that (1) EF functions as a bridge node connecting alpha ERD and the ADHD symptom network, and (2) motoric-type symptoms and EF deficits may constitute important nodes in the interplay between behavior/symptoms, cognition, and neurophysiological markers of ADHD.

## Supplemental Material

sj-docx-1-jad-10.1177_10870547241253999 – Supplemental material for A Network Approach to Understanding the Role of Executive Functioning and Alpha Oscillations in Inattention and Hyperactivity-Impulsivity Symptoms of ADHD Supplemental material, sj-docx-1-jad-10.1177_10870547241253999 for A Network Approach to Understanding the Role of Executive Functioning and Alpha Oscillations in Inattention and Hyperactivity-Impulsivity Symptoms of ADHD by Juan Diego Vera, René Freichel, Giorgia Michelini, Sandra K. Loo and Agatha Lenartowicz in Journal of Attention Disorders
