# Supplementary material for: A Network Approach to Understanding the Role of Executive Functioning and Alpha Oscillations in Inattention and Hyperactivity-Impulsivity Symptoms of ADHD
Source: J Atten Disord. 2024 May 26;28(10):1357–67. doi: 10.1177/10870547241253999 (PMC11292971; doi:10.1177/10870547241253999)
Supplement: sj-docx-1-jad-10.1177_10870547241253999 – Supplemental material for A Network Approach to Understanding the Role of Executive Functioning and Alpha Oscillations in Inattention and Hyperactivity-Impulsivity Symptoms of ADHD [file sj-docx-1-jad-10.1177_10870547241253999.docx]

**SUPPLEMENTARY INFORMATION**

***"*** ***A Network Approach to Understanding the Role of Executive Functioning and Alpha Oscillations in Inattention and Hyperactivity-Impulsivity Symptoms of ADHD. "***

[**Supplement 1. Harmonization of ADHD Symptoms** 2](#_Toc164345407)

[**Supplement 2. Harmonization of Executive Functioning Latent Construct** 5](#_Toc164345408)

[**Supplement 3. Edge Weights** 8](#_Toc164345409)

[**Supplement 4. Network Stability Analysis** 13](#_Toc164345410)

[**Supplement 5. Bridge Centrality Estimation** 13](#_Toc164345411)

[**Supplement 6. Sensitivity/Robustness Analyses** 14](#_Toc164345412)

[**Supplement 7. Separate subscale analyses** 15](#_Toc164345413)

[**Supplement 8. Distributions of symptom variables** 17](#_Toc164345414)

[**Supplementary References** 18](#_Toc164345415)

## **Supplement 1. Harmonization of ADHD Symptoms**

The SNAP and SWAN scales were harmonized to integrate both study samples ( Table S1). SNAP responses are ranked on a scale of 0 to 3, where 0 means that the problematic behavior is not at all present and 3 means that the problematic behavior is often present in the participant. The SWAN scores are on a 7-point scale (0 = far above average to 6 = far below average), in contrast to the SNAP, its scale range captures positive behaviors (Swanson et al., 2012). To harmonize the two scales, we converted all SNAP scores to the SWAN metric. If the SNAP scale score was 0, then it was converted to a 3 in the SWAN metric. If the scale score was a 1 in the SNAP, then it was converted to a 4 in the SWAN scale. If the SNAP scale score was a 2, then it was converted to a 5 in the SWAN. Finally, if the SNAP scale score was a 3, then it was converted to a 6 in the SWAN scale. The final symptom scale for the network analysis is a 7-point scale where 0 means that the participant does exceptionally well in a behavior and a score of 6 means that the behavior is problematic.

**Table S1**. *SWAN and SNAP Items and the corresponding Item Label and Subscale*

| Item Label | Item SWAN | Item SNAP | Subscale |  |
| --- | --- | --- | --- | --- |
| closatt | Give close attention to detail and avoid careless mistakes | Often fails to give close attention to details or makes careless mistakes in schoolwork or tasks | Inattention |  |
| sustain | Sustain attention on tasks or play activities | Often has difficulty sustaining attention in tasks or play activities | Inattention |  |
| listen | Listen when spoken to directly | Often does not seem to listen when spoken to directly | Inattention |  |
| instruct | Follow through on the instructions & finish schoolwork/chores | Often does not follow through on instructions and fails to finish schoolwork, chores, or duties | Inattention |  |
| org | Organize tasks and activities | Often has difficulty organizing tasks and activities | Inattention |  |
| susatt | Engage in tasks that require sustained mental effort | Often avoids, dislikes, or reluctantly engages in tasks requiring sustained mental effort | Inattention |  |
| loses | Keep track of things necessary for activities | Often loses things necessary for activities (e.g., toys, school assignments, pencils, or books) | Inattention |  |
| distract | Ignore extraneous stimuli | Often is distracted by extraneous stimuli | Inattention |  |
| forget | Remember daily activities | Often is forgetful in daily activities | Inattention | |
| fidget | Sit still (control movement of hands/feet or control squirming) | Often fidgets with hands or feet or squirms in seat | Hyperactivity/Impulsivity |  |
| seat | Stay seated (when required by class rules/social conventions) | Has difficulty staying seated according to classroom rules | Hyperactivity/Impulsivity |  |
| runs | Modulate motor activity (inhibit inappropriate running/climbing) | Often runs about or climbs excessively in situations in which it is inappropriate | Hyperactivity/Impulsivity |  |
| quiet | Play quietly (keep noise level reasonable) | Often has difficulty playing or engaging in leisure activities quietly | Hyperactivity/Impulsivity |  |
| motor | Settle down and rest (control constant activity) | Often is “on the go” or often acts as if “driven by a motor” | Hyperactivity/Impulsivity |  |
| talks | Modulate verbal activity (control excess talking) | Often talks excessively | Hyperactivity/Impulsivity |  |
| blurts | Reflect on questions (control blurting out answers) | Often blurts out answers before questions have been completed | Hyperactivity/Impulsivity |  |
| turn | Await turn (stand in line and take turns) | Often has difficulty awaiting turn | Hyperactivity/Impulsivity |  |
| interrupt | Enter into conversations and games (control interrupting/intruding) | Often interrupts or intrudes on others (e.g., butts into conversations/games) | Hyperactivity/Impulsivity |  |

## **Supplement 2.** **Harmonization of Executive Functioning Latent Construct**

Two datasets from two independent studies were integrated by following an analytical framework that is called Integrative Data Analysis or IDA (Curran et al., 2014; Curran & Hussong, 2009; Hussong et al., 2021). The strategy of IDA is to use psychometric modeling techniques to link the different measurements across studies and create comparable scales despite differences in assessment instruments and samples (Bainter & Curran, 2015; Curran et al., 2014). There are several approaches to data harmonization of cognitive tasks with its strengths and limitations, the most common approaches are standardization and latent variable modeling (Griffith et al., 2013). However, the use of latent variable modeling for harmonization has risen in popularity because it can address differences between samples by creating a common latent factor score that takes into account measurement and sample differences (Hussong et al., 2013; Tyrell et al., 2019).

The present study uses a latent variable approach called moderated nonlinear factor analysis (MNLFA; Bauer & Hussong, 2009) to create a common factor score across samples. The first step of MNLFA is to test dimensionality, we formally test the dimensionality of our item pool using exploratory factor analysis (EFA). An assumption for our analysis is that the scales used on each dataset are unidimensional. For this analysis, we first determined dimensionality within each of the datasets using an EFA, and then we improved model fit by correlating residuals from tasks that belong to the same battery. We retained 7 items to compose a one-factor solution that was clearly supported, with all items significantly loading on the factor for both datasets. The 7 items were: Trails letters time and switching times, Stroop color, word, and color-word score, and WAIS-IV digit forwards and digit backwards.

After establishing unidimensionality we used MNLFA to examine the effect of study membership on the parameters of the executive functioning latent model. Particularly we tested for differences in the factor means, factor variances, item intercepts (means), and item factor loadings (weights). To define the scale of the latent factor we fixed the conditional mean and variance of the factor (Executive Factor) to 0 and 1, respectively, then we estimated all item loadings and intercepts. We tested for item intercept and loading differences for each item in a sequential manner and examined for moderating effects of the covariates on the factor loading and intercept on each of the 7 variables.

We retained all significant covariate effects on the executive functioning factor (mean and variance) and items (thresholds and loadings), and then used the parameter estimates from the final model to produce maximum a posteriori (MAP) scores on the executive functioning latent model. The MAP score accounts for differences in items DIF, factor mean, and factor variance resulting from participants study membership.

**Table S2.** *Moderated nonlinear factor analysis (MNLFA) results and covariance effects*

| Item Covariance Effect | Intercept | Loading |
| --- | --- | --- |
| 1. Digit Forward | 6.937 | 0.561 |
| *Study 2* | ------- | ------- |
| 2. Digit Backward | 2.258 | 0.995 |
| *Study 2* | ------- | 0.542 |
| 3. Trails Letter | 0.149 | 0.378 |
| *Study 2* | ------- | ------- |
| 4. Trails Number-Letter | 0.500 | 0.300 |
| *Study 2* | ------- | ------- |
| 5. Stroop Color | 5.130 | 0.525 |
| *Study 2* | 1.005 | 0.423 |
| 6. Stroop Word | 5.649 | 0.403 |
| *Study 2* | 1.223 | ------- |
| 7. Stroop Color Word | 3.698 | 0.523 |
| *Study 2* | 0.112 | 0.413 |

*Note:* If there is an empy space (-------) it just means that there was not a significant difference between study membership for that parameter, therefore we did not estimate it in the final model.

The complete set of estimated effects for item intercepts and factor loadings are reported in Table S2. Results indicated that 4 of the 7 items showed differences in functioning across study membership, either by intercept or loadings. Some scales only showed DIF in the intercepts reflecting differences in probability of endorsing these scales at equivalent levels of executive functioning, and many of the other items show more complexity involving DIF on both item intercepts and factor loadings for study membership.

## **Supplement 3. Edge Weights**

**Table S3**. *Edge Weights For ADHD Symptom Network in Entire Sample (see Figure 1)*

|  | **closeatt** | **susatt** | **listen** | **instruct** | **org** | **avoid** | **loses** | **distract** | **forget** | **fidget** | **seat** | **runs** | **quiet** | **motor** | **talks** | **blurts** | **turn** | **interrupt** |
| --- | --- | --- | --- | --- | --- | --- | --- | --- | --- | --- | --- | --- | --- | --- | --- | --- | --- | --- |
| **closeatt** | 0 | 0.25 | 0.02 | 0.12 | 0.11 | 0.12 | 0.01 | 0.06 | 0.07 | 0 | 0 | -0.04 | 0 | 0 | 0 | 0 | 0 | 0 |
| **susatt** | 0.25 | 0 | 0.15 | 0.15 | 0.06 | 0.03 | 0 | 0.3 | 0.01 | 0.1 | 0 | -0.03 | 0 | 0 | 0.06 | 0 | 0 | 0 |
| **listen** | 0.02 | 0.15 | 0 | 0.12 | 0.05 | 0.11 | 0.05 | 0.08 | 0.1 | 0.04 | 0.09 | 0.02 | 0 | 0 | 0 | 0 | 0 | 0.08 |
| **instruct** | 0.12 | 0.15 | 0.12 | 0 | 0.25 | 0.13 | 0.16 | 0 | 0.11 | 0 | 0.02 | 0 | 0 | 0 | 0 | 0 | 0 | 0 |
| **org** | 0.11 | 0.06 | 0.05 | 0.25 | 0 | 0.13 | 0.17 | 0.1 | 0.17 | 0.05 | 0 | 0 | 0 | 0 | 0.02 | 0 | 0 | 0.01 |
| **avoid** | 0.12 | 0.03 | 0.11 | 0.13 | 0.13 | 0 | 0.13 | 0.14 | 0 | 0 | 0.05 | 0 | 0.01 | 0 | 0 | 0 | 0 | 0.02 |
| **loses** | 0.01 | 0 | 0.05 | 0.16 | 0.17 | 0.13 | 0 | 0.01 | 0.35 | 0.01 | 0.01 | 0.06 | 0 | 0 | -0.02 | 0 | 0 | 0 |
| **distract** | 0.06 | 0.3 | 0.08 | 0 | 0.1 | 0.14 | 0.01 | 0 | 0.11 | 0.11 | 0.01 | -0.01 | 0 | 0.02 | 0.01 | 0 | 0 | 0 |
| **forget** | 0.07 | 0.01 | 0.1 | 0.11 | 0.17 | 0 | 0.35 | 0.11 | 0 | 0.03 | 0.03 | 0 | 0 | 0 | 0 | 0 | 0 | 0 |
| **fidget** | 0 | 0.1 | 0.04 | 0 | 0.05 | 0 | 0.01 | 0.11 | 0.03 | 0 | 0.29 | 0.04 | 0.1 | 0.03 | 0.02 | 0.04 | 0.03 | 0.01 |
| **seat** | 0 | 0 | 0.09 | 0.02 | 0 | 0.05 | 0.01 | 0.01 | 0.03 | 0.29 | 0 | 0.15 | 0.11 | 0.16 | 0 | 0 | 0.08 | 0.04 |
| **runs** | -0.04 | -0.03 | 0.02 | 0 | 0 | 0 | 0.06 | -0.01 | 0 | 0.04 | 0.15 | 0 | 0.23 | 0.29 | 0 | 0 | 0.04 | 0.08 |
| **quiet** | 0 | 0 | 0 | 0 | 0 | 0.01 | 0 | 0 | 0 | 0.1 | 0.11 | 0.23 | 0 | 0.24 | 0.06 | 0.02 | 0.07 | 0.08 |
| **motor** | 0 | 0 | 0 | 0 | 0 | 0 | 0 | 0.02 | 0 | 0.03 | 0.16 | 0.29 | 0.24 | 0 | 0.17 | 0.04 | 0.09 | 0 |
| **talks** | 0 | 0.06 | 0 | 0 | 0.02 | 0 | -0.02 | 0.01 | 0 | 0.02 | 0 | 0 | 0.06 | 0.17 | 0 | 0.34 | 0.06 | 0.09 |
| **blurts** | 0 | 0 | 0 | 0 | 0 | 0 | 0 | 0 | 0 | 0.04 | 0 | 0 | 0.02 | 0.04 | 0.34 | 0 | 0.3 | 0.25 |
| **turn** | 0 | 0 | 0 | 0 | 0 | 0 | 0 | 0 | 0 | 0.03 | 0.08 | 0.04 | 0.07 | 0.09 | 0.06 | 0.3 | 0 | 0.37 |
| **interrupt** | 0 | 0 | 0.08 | 0 | 0.01 | 0.02 | 0 | 0 | 0 | 0.01 | 0.04 | 0.08 | 0.08 | 0 | 0.09 | 0.25 | 0.37 | 0 |

**Table S4**. *Edge Weights For EF-Alpha-Inattention Symptom Network in Entire Sample (see Figure 3)*

|  | **closeatt** | **susatt** | **listen** | **instruct** | **org** | **avoid** | **loses** | **distract** | **forget** | **alpha_occ** | **EF** |
| --- | --- | --- | --- | --- | --- | --- | --- | --- | --- | --- | --- |
| **closeatt** | 0 | 0.23 | 0.03 | 0.09 | 0.09 | 0.13 | 0.04 | 0.05 | 0.05 | 0 | 0 |
| **susatt** | 0.23 | 0 | 0.19 | 0.15 | 0.08 | 0.06 | 0 | 0.32 | 0.02 | 0 | 0 |
| **listen** | 0.03 | 0.19 | 0 | 0.13 | 0.07 | 0.12 | 0.07 | 0.07 | 0.14 | 0 | -0.09 |
| **instruct** | 0.09 | 0.15 | 0.13 | 0 | 0.23 | 0.16 | 0.16 | 0 | 0.11 | 0 | 0 |
| **org** | 0.09 | 0.08 | 0.07 | 0.23 | 0 | 0.14 | 0.17 | 0.13 | 0.19 | 0 | 0 |
| **avoid** | 0.13 | 0.06 | 0.12 | 0.16 | 0.14 | 0 | 0.13 | 0.13 | 0.01 | 0 | 0 |
| **loses** | 0.04 | 0 | 0.07 | 0.16 | 0.17 | 0.13 | 0 | 0.03 | 0.34 | 0.03 | 0 |
| **distract** | 0.05 | 0.32 | 0.07 | 0 | 0.13 | 0.13 | 0.03 | 0 | 0.13 | 0 | 0 |
| **forget** | 0.05 | 0.02 | 0.14 | 0.11 | 0.19 | 0.01 | 0.34 | 0.13 | 0 | 0 | 0 |
| **alpha_occ** | 0 | 0 | 0 | 0 | 0 | 0 | 0.03 | 0 | 0 | 0 | -0.29 |
| **EF** | 0 | 0 | -0.09 | 0 | 0 | 0 | 0 | 0 | 0 | -0.29 | 0 |

**Table S5**. *Edge Weights For EF-Alpha-Hyperactivity-Impulsivity Symptoms Network (see Figure 4)*

|  | **fidget** | **seat** | **runs** | **quiet** | **motor** | **talks** | **blurts** | **turn** | **interrupt** | **alpha_occ** | **EF** |
| --- | --- | --- | --- | --- | --- | --- | --- | --- | --- | --- | --- |
| **fidget** | 0 | 0.39 | 0.05 | 0.11 | 0.03 | 0.06 | 0.04 | 0.02 | 0.07 | 0 | -0.02 |
| **seat** | 0.39 | 0 | 0.13 | 0.11 | 0.21 | 0 | 0.01 | 0.08 | 0.05 | 0 | -0.07 |
| **runs** | 0.05 | 0.13 | 0 | 0.21 | 0.27 | 0 | 0 | 0.05 | 0.08 | 0 | -0.11 |
| **quiet** | 0.11 | 0.11 | 0.21 | 0 | 0.23 | 0.04 | 0.03 | 0.07 | 0.1 | 0 | 0 |
| **motor** | 0.03 | 0.21 | 0.27 | 0.23 | 0 | 0.23 | 0.02 | 0.1 | 0 | -0.02 | 0 |
| **talks** | 0.06 | 0 | 0 | 0.04 | 0.23 | 0 | 0.37 | 0.03 | 0.09 | 0 | 0 |
| **blurts** | 0.04 | 0.01 | 0 | 0.03 | 0.02 | 0.37 | 0 | 0.31 | 0.22 | 0.02 | 0 |
| **turn** | 0.02 | 0.08 | 0.05 | 0.07 | 0.1 | 0.03 | 0.31 | 0 | 0.37 | 0 | 0 |
| **interrupt** | 0.07 | 0.05 | 0.08 | 0.1 | 0 | 0.09 | 0.22 | 0.37 | 0 | 0 | -0.03 |
| **alpha_occ** | 0 | 0 | 0 | 0 | -0.02 | 0 | 0.02 | 0 | 0 | 0 | -0.29 |
| **EF** | -0.02 | -0.07 | -0.11 | 0 | 0 | 0 | 0 | 0 | -0.03 | -0.29 | 0 |

**Table S6**. *Edge Weights For EF-Alpha-ADHD Symptom Network in Entire Sample (see Figure 5)*

|  | **closeatt** | **susatt** | **listen** | **instruct** | **org** | **avoid** | **loses** | **distract** | **forget** | **fidget** | **seat** | **runs** | **quiet** | **motor** | **talks** | **blurts** | **turn** | **interrupt** | **alpha_occ** | **EF** |
| --- | --- | --- | --- | --- | --- | --- | --- | --- | --- | --- | --- | --- | --- | --- | --- | --- | --- | --- | --- | --- |
| **closeatt** | 0 | 0.23 | 0 | 0.09 | 0.09 | 0.13 | 0 | 0 | 0 | 0 | 0 | 0 | 0 | 0 | 0 | 0 | 0 | 0 | 0 | 0 |
| **susatt** | 0.23 | 0 | 0.16 | 0.14 | 0.06 | 0 | 0 | 0.3 | 0 | 0.12 | 0 | 0 | 0 | 0 | 0.07 | 0 | 0 | 0 | 0 | 0 |
| **listen** | 0 | 0.16 | 0 | 0.12 | 0 | 0.09 | 0 | 0 | 0.12 | 0 | 0.07 | 0 | 0 | 0 | 0 | 0 | 0 | 0.09 | 0 | 0 |
| **instruct** | 0.09 | 0.14 | 0.12 | 0 | 0.23 | 0.15 | 0.16 | 0 | 0.1 | 0 | 0 | 0 | 0 | 0 | 0 | 0 | 0 | 0 | 0 | 0 |
| **org** | 0.09 | 0.06 | 0 | 0.23 | 0 | 0.14 | 0.17 | 0.12 | 0.19 | 0 | 0 | 0 | 0 | 0 | 0 | 0 | 0 | 0 | 0 | 0 |
| **avoid** | 0.13 | 0 | 0.09 | 0.15 | 0.14 | 0 | 0.12 | 0.12 | 0 | 0 | 0 | 0 | 0 | 0 | 0 | 0 | -0.07 | 0.07 | 0 | 0 |
| **loses** | 0 | 0 | 0 | 0.16 | 0.17 | 0.12 | 0 | 0 | 0.35 | 0 | 0 | 0.08 | 0 | 0 | 0 | 0 | 0 | 0 | 0 | 0 |
| **distract** | 0 | 0.3 | 0 | 0 | 0.12 | 0.12 | 0 | 0 | 0.12 | 0.1 | 0 | 0 | 0 | 0 | 0 | 0 | 0 | 0 | 0 | 0 |
| **forget** | 0 | 0 | 0.12 | 0.1 | 0.19 | 0 | 0.35 | 0.12 | 0 | 0 | 0.06 | 0 | 0 | 0 | 0 | 0 | 0 | 0 | 0 | 0 |
| **fidget** | 0 | 0.12 | 0 | 0 | 0 | 0 | 0 | 0.1 | 0 | 0 | 0.3 | 0 | 0.1 | 0 | 0 | 0 | 0 | 0 | 0 | 0 |
| **seat** | 0 | 0 | 0.07 | 0 | 0 | 0 | 0 | 0 | 0.06 | 0.3 | 0 | 0.11 | 0.11 | 0.19 | 0 | 0 | 0.09 | 0 | 0 | -0.1 |
| **runs** | 0 | 0 | 0 | 0 | 0 | 0 | 0.08 | 0 | 0 | 0 | 0.11 | 0 | 0.21 | 0.28 | 0 | 0 | 0 | 0.06 | 0 | -0.12 |
| **quiet** | 0 | 0 | 0 | 0 | 0 | 0 | 0 | 0 | 0 | 0.1 | 0.11 | 0.21 | 0 | 0.24 | 0 | 0 | 0.07 | 0.09 | 0 | 0 |
| **motor** | 0 | 0 | 0 | 0 | 0 | 0 | 0 | 0 | 0 | 0 | 0.19 | 0.28 | 0.24 | 0 | 0.23 | 0 | 0.1 | 0 | 0 | 0 |
| **talks** | 0 | 0.07 | 0 | 0 | 0 | 0 | 0 | 0 | 0 | 0 | 0 | 0 | 0 | 0.23 | 0 | 0.38 | 0 | 0.09 | 0 | 0 |
| **blurts** | 0 | 0 | 0 | 0 | 0 | 0 | 0 | 0 | 0 | 0 | 0 | 0 | 0 | 0 | 0.38 | 0 | 0.32 | 0.21 | 0 | 0 |
| **turn** | 0 | 0 | 0 | 0 | 0 | -0.07 | 0 | 0 | 0 | 0 | 0.09 | 0 | 0.07 | 0.1 | 0 | 0.32 | 0 | 0.38 | 0 | 0 |
| **interrupt** | 0 | 0 | 0.09 | 0 | 0 | 0.07 | 0 | 0 | 0 | 0 | 0 | 0.06 | 0.09 | 0 | 0.09 | 0.21 | 0.38 | 0 | 0 | 0 |
| **alpha_occ** | 0 | 0 | 0 | 0 | 0 | 0 | 0 | 0 | 0 | 0 | 0 | 0 | 0 | 0 | 0 | 0 | 0 | 0 | 0 | -0.31 |
| **EF** | 0 | 0 | 0 | 0 | 0 | 0 | 0 | 0 | 0 | 0 | -0.1 | -0.12 | 0 | 0 | 0 | 0 | 0 | 0 | -0.31 | 0 |

## **Supplement 4. Network Stability Analysis**

**Table S7**. *Correlation Stability Coefficients for All Networks.*

| Network | Edge weights | Strength centrality |
| --- | --- | --- |
| ADHD symptoms | 0.75 | 0.52 |
| ADHD symptoms – EF - Alpha | 0.75 | 0.75 |
| Hyperactivity-Impulsivity Symptoms – EF - Alpha | 0.75 | 0.67 |
| Inattention Symptoms – EF - Alpha | 0.67 | 0,59 |

## **Supplement 5. Bridge Centrality Estimation**

**Figure S1**

*Bridge Centrality Strength Estimates For All Nodes in Main ADHD Symptom Network*

**
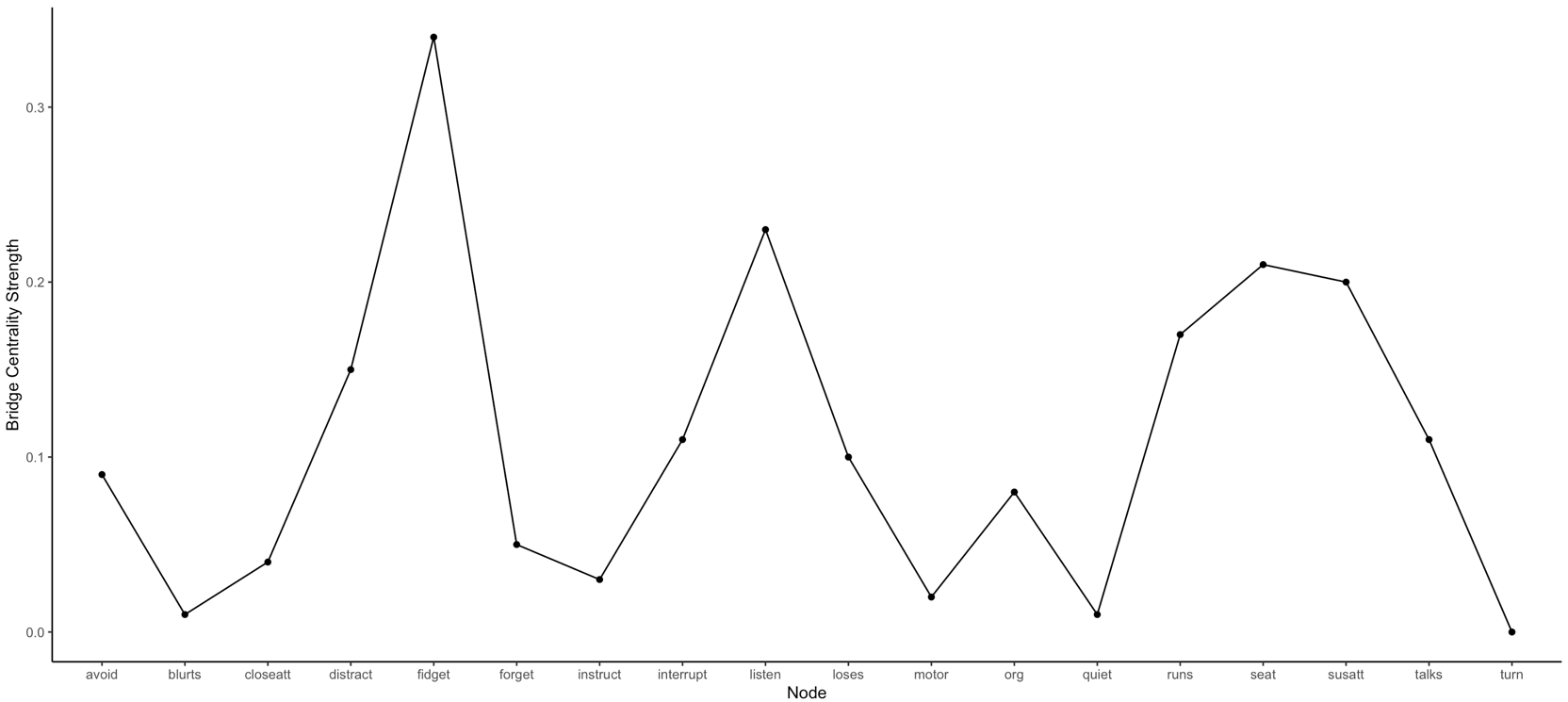
**

## **Supplement 6. Sensitivity/Robustness Analyses**

Network comparison tests (van Borkulo et al., 2022) were used for additional sensitivity/ robustness analyses to test potential differences in the network structure for age and sex groups. These analyses refer to the core network that includes EF, alpha, inattentive, and hyperactive-impulsive symptoms. This permutation-based hypothesis examined differences in the overall network structure and global network strength. First, we used a median split to form two age groups, then we estimated separate networks in these groups and finally compared the networks using network comparison tests. Similarly, we compared the networks estimated separately for men and women.

The comparison tests were done using the NetworkComparisonTest package (Borkulo et al., 2023) with 1000 iterations (permutations). These robustness analyses showed no significant differences between age groups in the network structure (p = 0.43, M = 0.20) or global network strength (p = 0.83, S = 0.04). Also, we found no significant differences between men and women with respect to the overall network structure (p = 0.12, M = 0.33) or global network strength (p = 0.54, S = 0.48).

## **Supplement 7. Separate subscale analyses**

**Figure S2**

*Inattention – Alpha – EF Network*


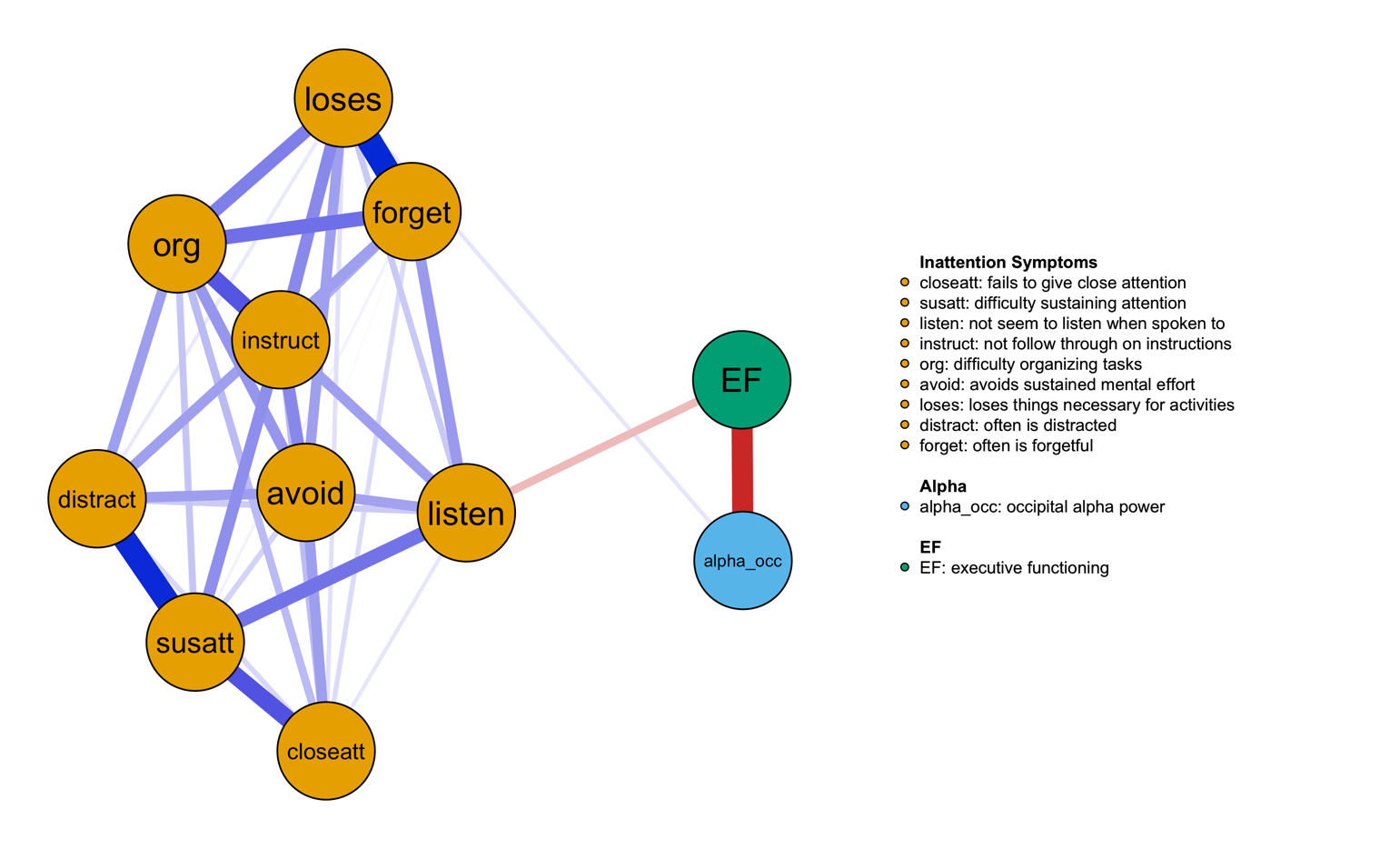


**Figure S3**

*Hyperactivity-impulsivity – Alpha – EF Network*

**
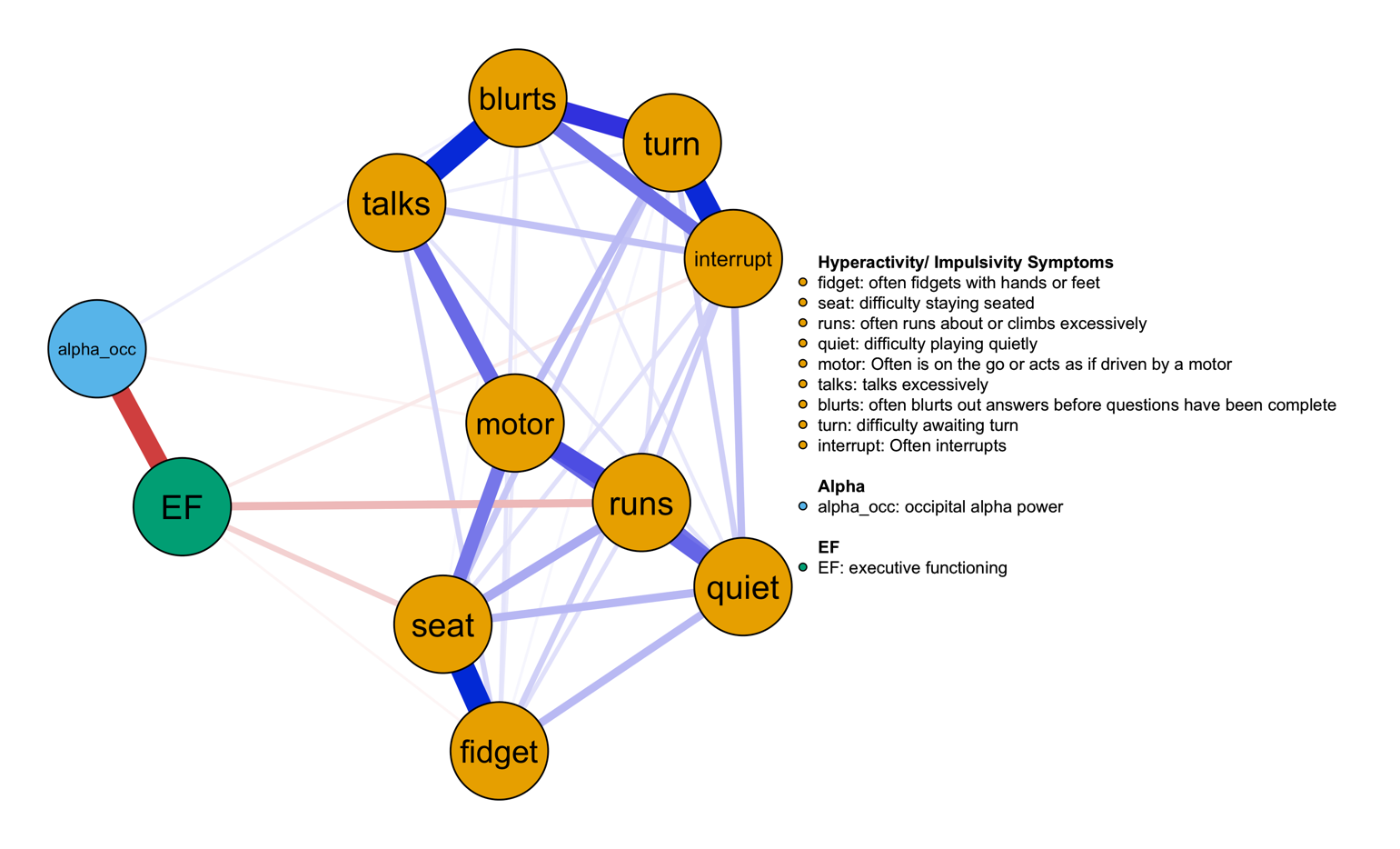
**

## **Supplement 8. Distributions of symptom variables**

**Figure S4**

Distributions of symptom measures


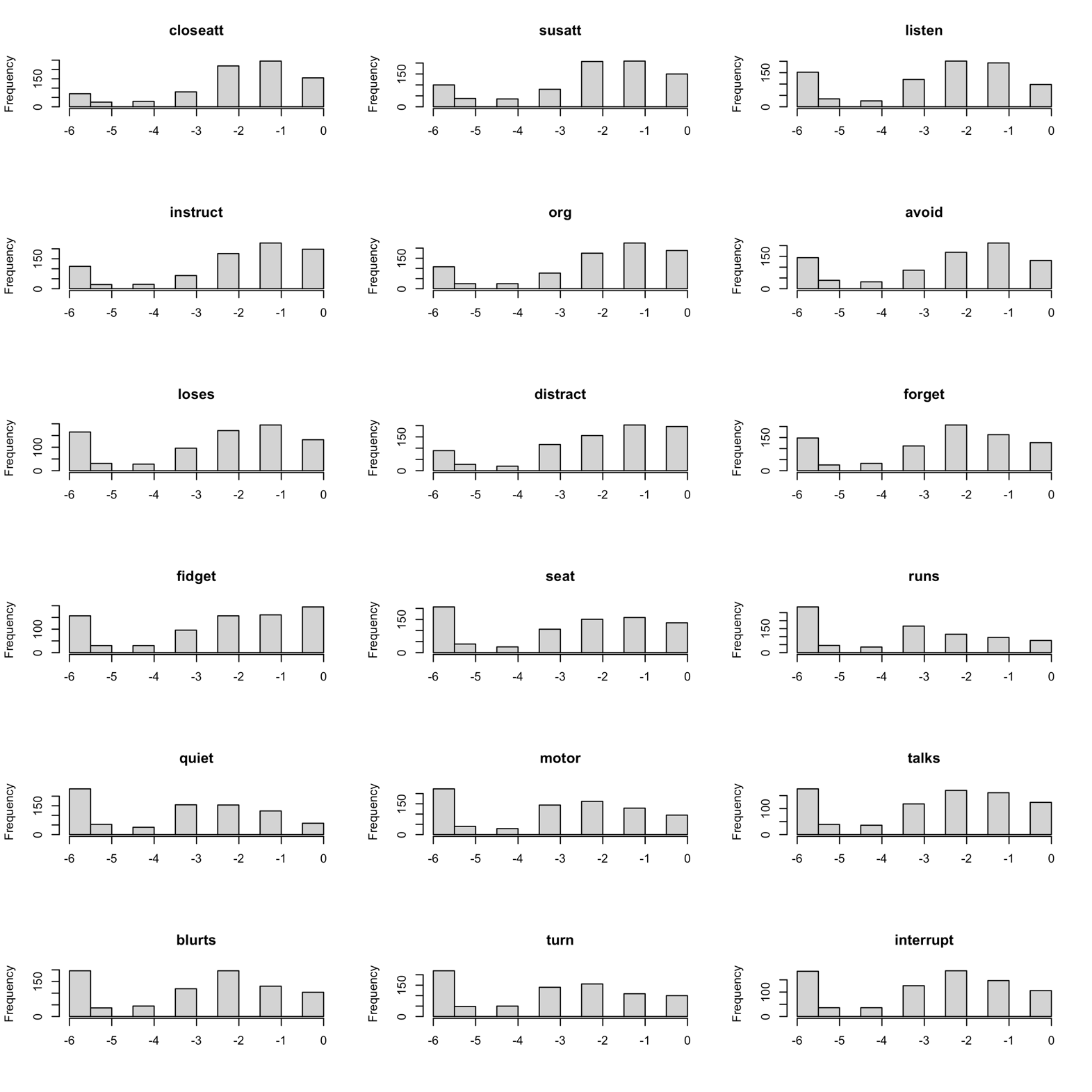


## **Supplementary References**

Bainter, S. A., & Curran, P. J. (2015). Advantages of integrative data analysis for developmental research. *Journal of Cognition and Development*, *16*(1), 1–10.

Bauer, D. J., & Hussong, A. M. (2009). Psychometric approaches for developing commensurate measures across independent studies: Traditional and new models. *Psychological Methods*, *14*(2), 101.

Borkulo, C. van, Epskamp, S., Jones, P., Haslbeck, J., Millner, A., Huth, K., & Bergh, D. van den. (2023). *NetworkComparisonTest: Statistical Comparison of Two Networks Based on Several Invariance Measures* (2.2.2) [Computer software]. https://cran.r-project.org/web/packages/NetworkComparisonTest/index.html

Curran, P. J., & Hussong, A. M. (2009). Integrative data analysis: The simultaneous analysis of multiple data sets. *Psychological Methods*, *14*(2), 81.

Curran, P. J., McGinley, J. S., Bauer, D. J., Hussong, A. M., Burns, A., Chassin, L., Sher, K., & Zucker, R. (2014). A moderated nonlinear factor model for the development of commensurate measures in integrative data analysis. *Multivariate Behavioral Research*, *49*(3), 214–231.

Griffith, L., van den Heuvel, E., Fortier, I., Hofer, S., Raina, P., Sohel, N., Payette, H., Wolfson, C., & Belleville, S. (2013). *Harmonization of cognitive measures in individual participant data and aggregate data meta-analysis*.

Hussong, A. M., Bauer, D. J., Giordano, M. L., & Curran, P. J. (2021). Harmonizing altered measures in integrative data analysis: A methods analogue study. *Behavior Research Methods*, *53*(3), 1031–1045.

Hussong, A. M., Curran, P. J., & Bauer, D. J. (2013). Integrative data analysis in clinical psychology research. *Annual Review of Clinical Psychology*, *9*, 61.

Swanson, J. M., Schuck, S., Porter, M. M., Carlson, C., Hartman, C. A., Sergeant, J. A., Clevenger, W., Wasdell, M., McCleary, R., & Lakes, K. (2012). Categorical and dimensional definitions and evaluations of symptoms of ADHD: history of the SNAP and the SWAN rating scales. *The International Journal of Educational and Psychological Assessment*, *10*(1), 51.

Tyrell, F. A., Yates, T. M., Widaman, K. F., Reynolds, C. A., & Fabricius, W. V. (2019). Data harmonization: Establishing measurement invariance across different assessments of the same construct across adolescence. *Journal of Clinical Child & Adolescent Psychology*, *48*(4), 555–567.

van Borkulo, C. D., van Bork, R., Boschloo, L., Kossakowski, J. J., Tio, P., Schoevers, R. A., Borsboom, D., & Waldorp, L. J. (2022). Comparing network structures on three aspects: A permutation test. *Psychological Methods*. https://doi.org/10.1037/met0000476
